# Deterministic generation of indistinguishable photons in a cluster state

**DOI:** 10.1038/s41566-022-01152-2

**Published:** 2023-02-09

**Authors:** Dan Cogan, Zu-En Su, Oded Kenneth, David Gershoni

**Affiliations:** The Physics Department and the Solid State Institute, Technion — Israel Institute of Technology, Haifa, Israel

**Keywords:** Quantum optics, Quantum information

## Abstract

Entanglement between particles is a basic concept of quantum sciences. The ability to produce entangled particles in a controllable manner is essential for any quantum technology. Entanglement between light particles (photons) is particularly crucial for quantum communication due to light’s non-interactive nature and long-lasting coherence. Resources producing entangled multiphoton cluster states will enable communication between remote quantum nodes, as the inbuilt redundancy of cluster photons allows for repeated local measurements—compensating for losses and probabilistic Bell measurements. For feasible applications, the cluster generation should be fast, deterministic and, most importantly, its photons indistinguishable, which will allow measurements and fusion of clusters by interfering photons. Here, using periodic excitation of a semiconductor quantum-dot-confined spin, we demonstrate a multi-indistinguishable photon cluster, featuring a continuously generated string of photons at deterministic gigahertz generation rates, and an optimized entanglement length of about ten photons. The indistinguishability of the photons opens up new possibilities for scaling up the cluster’s dimensionality by fusion, thus building graph states suited for measurement-based photonic quantum computers and all-photonic quantum repeaters.

## Abstract (web summary)

A continuous string of indistinguishable photons entangled in a cluster state is generated on demand from an InAs/GaAs quantum dot. The confined heavy-hole spin is used as an entangler. Under an externally tuned magnetic field, an optimized characteristic entanglement decay length of about ten photons is obtained.

## Main

Entanglement is a fundamental concept of quantum mechanics^1^. The ability to generate, impose, control and distribute entanglement is crucial for quantum technologies, quantum information processing and, in particular, quantum networking^2^. The non-interacting nature of photons, which protects them from dephasing, makes them a natural carrier for quantum communication; however, as photons do not mutually interact, their use for entanglement distribution is challenging. Zeilinger, Pan and co-workers introduced second-order interference between indistinguishable photons as a way to partially overcome this problem^3,4^. Photon indistinguishability (ID) is therefore a major prerequisite for entanglement distribution and photonic quantum repeaters. The unavoidable photon loss that exponentially limits the success probability of any communication protocol with distance, and the probabilistic nature of the interference remain as barriers that prevent the development of a full-fledged quantum network. Overcoming the exponential loss is possible using the concept of quantum repeaters^5^, in which entanglement swapping and purification performed at intermediate nodes enable entanglement distribution between distant nodes with photons^6^.

Inspired by measurement-based quantum computation^7,8^, Zwerger and co-workers introduced the idea of measurement-based quantum repeaters^9^. This network architecture uses multiphoton entangled states—cluster or graph states^10–13^—that can be distributed and mutually connected through Bell measurements. Azuma and co-workers^14^ recently extended this idea by making the repeater graph exclusively photonic, thereby removing the need for long-lived quantum memory. Graph states provide redundancy against photon loss and the probabilistic nature of photonic Bell measurements: if the first measurement fails, more trials will substantially increase the probability of success. An essential requirement from such graph states is that the photons be indistinguishable. Developing devices that are capable of deterministically producing indistinguishable photonic graph states is therefore an important scientific and technological challenge to overcome^15–19^.

Single and entangled photons can be generated via the spontaneous emission of an optical transition of an atom or an atom-like quantum emitter^1,20^. Artificial atoms such as semiconductor quantum dots (QDs) have demonstrated tremendous performance as they can be incorporated into electro-optical devices, which dovetail with the contemporary semiconductor-based industry. Semiconductor QD-based devices have shown high efficiencies and photon emission rates (see ref. ^21^ for a review), placing them as the forerunner of all physical systems considered for generating quantum light^9,12,14,15,22^.

Of particular relevance to this work is Lindner and Rudolph’s^23^ proposal to generate one-dimensional photonic cluster states using semiconductor QDs. Their scheme uses a single QD-confined electron spin precessing in a magnetic field while driven by a temporal sequence of laser pulses. Following excitation of the QD, a single photon is deterministically emitted, and its polarization is entangled with the state of the QD-confined spin^24^. This process repeats many times to generate a large one-dimensional cluster of entangled photons.

The entanglement robustness of such a photonic cluster is mainly determined by the ratio between the optical transition radiative and spin precession rates^25^, and by the coherence properties of the QD spin^26,27^. The ID between the emitted photons is mainly determined by the nature of the optical transition, which results in photon emission^28^. Schwartz and colleagues demonstrated a modification of this proposal by generating a one-dimensional cluster state based on a QD-confined dark exciton^29^ as a photon entangler, and by characterizing the cluster entanglement length^25^. During generation, the emitted photons leave the dark exciton in an excited state, having a shorter lifetime than the radiative time. Consequently, the instability of the final state introduces spectral broadening to the energy profiles of the photons, resulting in non-identical photonic emission.

Here we present an on-demand, continuously generated string of indistinguishable photons entangled in a cluster state. We use the heavy-hole (HH) spin^26,30,31^ as an entangler, utilizing the fact that the HH is left in a stable ground state after photon emission; thus, as we show below, the emitted single photons are highly indistinguishable. Moreover, the HH has a half-integer spin; therefore, in the absence of external magnetic field, its two spin states are Kramers’ degenerate and its precession rate vanishes. Fine-tuning of the external field’s strength provides means for controlling the spin precession and photon emission rates, as well as optimization of the entanglement robustness in the cluster state.

**Fig. 1:**
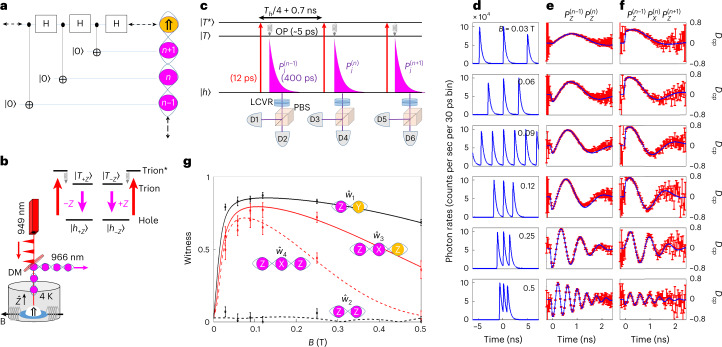
The measured cluster witnesses. **a**, Ideal description of the cluster state generation using sequential Hadamard and CNOT gates. **b**, A schematic of the QD-based device. DM represents a dichroic mirror and ${\overrightarrow{B}}=B{\hat{x}}$ the externally applied magnetic field. The inset shows the polarization selection rules for the optical transition. The terms $\left\vert h\right\rangle$ ($\left\vert \Uparrow \right\rangle$), $\left\vert T\right\rangle$ and $\left\vert {T}^{* }\right\rangle$ represent the HH, trion and excited trion states, respectively; ±*Z* represents the photon’s circular polarization along the optical axis and also the spin state’s direction. Red (pink) arrows represent laser pulses (emitted photons), whereas spiral arrows represent optical-phonon-assisted transitions. **c**, Schematic description of the QD’s spin-configurations optical transitions and their detection used in the measurements. The pink exponentials represent emitted single photons; *T*_*h*_ is the HH spin precession period. We detect two and three consecutive photons from the cluster and project their polarization using liquid crystal variable retarders (LCVR) and polarized beam splitters (PBS); ${P}_{j}^{(i)}$ represents the polarization of the *i*th photon in the cluster, projected onto the *j*th polarization state. **d**, Time-resolved single photon rate measurements for various magnetic fields. At ~0.09 T, the HH precession is such that the appropriate excitation rate is exactly six times faster than the laser repetition rate (~76 MHz), resulting in deterministic generation of a continuous string of photons. **e**,**f**, Two- (**e**) and three-photon (**f**) correlations; red marks are the time-resolved measurement of the last correlated photon’s *D*_cp_ as a function of time from the second (third) pulse. These *D*_cp_ values are correlated with the previous detection of an emitted photon in **e** and two photons in **f**. Blue lines represent the best fitted central-spin-evolution model for the positive trion^26,35^. **g**, The measured (error bars) and calculated (coloured lines) cluster state witnesses as deduced from the fits in **e** and **f** versus the in-plane magnetic field strength. Strings of polarized photons (pink circles) and spin (yellow circles) describe the witnesses; ${\hat{w}}_{1}$ and ${\hat{w}}_{3}$ are deduced from the polarization of the correlated detection of two- and three-photons, as shown in **e** and **f**, respectively; ${\hat{w}}_{2}$ and ${\hat{w}}_{4}$ are deduced by temporally integrating these responses over one radiative lifetime. The error bars represent statistical 1 s.d. uncertainties deduced from a temporally integrated total number of about 500,000 two- and 50,000 three-sequential photons detection events.

We follow ref. ^23^, as schematically described in Fig. 1a, by applying a periodic sequence of controlled-not (CNOT) and Hadamard gates on the HH for producing the cluster state. Figure 1b,c schematically describes the QD-based device, and the experimental system for generating the cluster and its characterization, respectively. Each laser pulse (red upwards arrows) excites the confined HH ($\left\vert h\right\rangle =\left\vert \Uparrow \right\rangle$) to the excited-positive trion state ($\left\vert {T}^{* }\right\rangle =\left\vert \Uparrow \Downarrow \uparrow \right\rangle$*), in which the electron resides in its respective second energy level. The electron decays to the trion ground ($\left\vert T\right\rangle$) level within about 5 ps by emitting a spin-preserving optical phonon^32,33^. The trion then recombines within about 400 ps, by emitting a photon (marked pink), leaving the HH at its ground level ($\left\vert h\right\rangle$). A dichroic mirror steers the emitted photons to the detectors.

Both the HH and the positive trion act as spin qubits^34^. The selection rules for the optical transitions (see Fig. 1b) associated with the excitation and emission result in entanglement between the photon polarization and the HH spin polarization (see the Supplementary Information). Each excitation–emission step is therefore an actual realization of a CNOT gate between the spin and the photon qubits. We realize the Hadamard gate on the spin qubit by timing the excitations to the field-induced HH precession, such that after each excitation and photon emission, the spin acquires a π/2 phase before it is excited again. A sequence of resonantly tuned, linearly polarized laser π-pulses experimentally realizes the deterministic generation of the cluster state (Fig. 1c). Each pulse results in the addition of an entangled photon to the cluster state. As the cluster protocol is made by repeating an identical cycle, composed of excitation, emission and spin rotation, one can characterize the entire cluster state by mapping the process of this identical cycle. We perform full process tomography of the identical cycle for an externally applied magnetic field of 0.12 T (see the Supplementary Information). We measured four cluster witnesses for all of the other magnetic field strengths, as described below. For these purposes, it is enough to apply three cycles of the protocol and to detect the three photons, which the QD emits as a result^25^. For the photon detection and their polarization projection, we use six single-photon detectors in the set-up as described in Fig. 1c. In Fig. 1d we present the time-resolved single-photon detection rates as recorded by each of these six detectors for six different magnetic field strengths. For each field strength, we timed the excitation to match one-quarter of the HH spin precession period plus a small addition compensating for the finite trion’s radiative time. At the specific field strength of about 0.09 T, the HH precession is such that the appropriate excitation rate is exactly six-times faster than the laser repetition rate (~76 MHz). Under these finely tuned synchronization conditions, the device continuously generates a string of entangled photons, thereby realizing the original proposal of ref. ^23^, albeit by using the HH rather than electron spin.

We then consider all of the events in which two and three photons are detected sequentially. In Fig. 1e (Fig. 1f) we present the time-resolved degree-of-circular-polarization (*D*_cp_) measurements of the second (third) sequentially detected photon. We use these measurements to monitor the trion’s spin evolution during its radiative decay and hence determine the HH spin state before excitation^35,36^. We note that these time-resolved measurements are only used as characterization method enabling independent hole spin tomography. They are not part of the cluster generation protocol. We extract the following four cluster state witnesses from these measurements: ${\hat{w}}_{1}={P}_{Z}^{(n-1)}{S}_{Y}$, ${\hat{w}}_{2}={P}_{Z}^{(n-1)}{P}_{Z}^{(n)}$, ${\hat{w}}_{3}={P}_{Z}^{(n-1)}{P}_{X}^{(n)}{S}_{Z}$ and ${\hat{w}}_{4}={P}_{Z}^{(n-1)}{P}_{X}^{(n)}{P}_{Z}^{(n+1)}$, where ${P}_{j}^{(i)}$ represents the polarization of the *i*th photon in the string, projected onto the *jt*h polarization state; and *S*_*j*_ is the polarization of the HH spin, projected onto the *j*th state. We note that ${\hat{w}}_{1}$ and ${\hat{w}}_{3}$ are directly extracted from the polarization of correlated detection of two- and three-photons, shown in Fig. 1e,f, respectively, whereas ${\hat{w}}_{2}$ and ${\hat{w}}_{4}$ are extracted from the temporal integration of these measurements over the photon’s lifetime. For an ideal cluster state (containing ideal CNOT and Hadamard gates), ${\hat{w}}_{1},{\hat{w}}_{3}$ and ${\hat{w}}_{4}$ equal 1, whereas ${\hat{w}}_{2}$ equals 0.

In Fig. 1g we present the four measured witnesses as a function of the magnetic field strength. The solid lines are the calculated witnesses using our state-evolution-model (see the Suppementary Information). The parameters used for the model calculations were independently measured and listed in ref. ^36^. A good quantitative agreement between the measured data and the calculations is obtained.

**Fig. 2:**
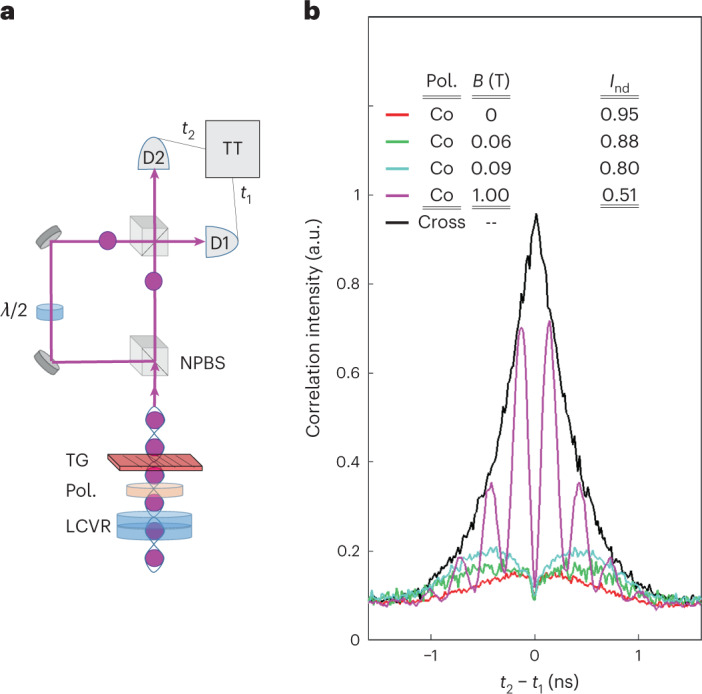
Photon ID. **a**, The interferometer set-up. Two consecutive cluster photons interfere on a non-polarizing beam splitter (NPBS). Two detectors on the output ports register their detection times, *t*_1_ and *t*_2_, respectively. A half-wave plate (*λ*/2) is used to select the photons’ relative polarization state between co- and cross-linear polarization. A pair of LCVRs and a linear polarizer (Pol.) project the photons’ polarization, and a transmission grating (TG) filters their energy. **b**, ID measurements (averaged over both rectilinear polarizations H and V) between two consecutive cluster photons for various externally applied magnetic fields. The solid lines represent the measured second-order correlation function *g*^(2)^(*t*_2_ − *t*_1_) using 50,000 two-sequential photon correlation events. In each period (13 ns apart) there are five correlation peaks^37^. Only the central peak, which results from the interference of the two photons, is shown. The other four peaks form the measured background. As they are polarization independent, they can be straightforwardly subtracted from the measured interference. The measured ID is then defined as *I*_nd_ = 1 − *A*_co_/*A*_cross_, where *A*_co_ (*A*_cross_) is the area under the co (cross)-polarized correlation function, represented by the coloured (black) solid lines.

We now turn to measure the ID between two sequential photons in the cluster state by using the same set-up used by Hong, Ou and Mandel^4,37^, as illustrated in Fig. 2a. The ID is given by the ratio between the second-order interference of co- to cross-polarized photons, as displayed in Fig. 2b for various magnetic fields. At zero magnetic field, the cluster photons’ ID amounts to 95%. The ID between photons emitted from a quantum source of single photons depends on the temporal stability of the initial and final states^28^. Here the final state is the temporally stable ground state of the HH, hence the high ID of the cluster photons (see the Supplementary Information).

The externally applied magnetic field reduces the ID of the cluster photons. As the field increases, the ID decreases due to the field-induced spectral broadening. At large fields, when the Zeeman splitting surpasses the radiative linewidth (see the Supplementary Information), the averaged (over both polarizations) ID drops to 50% and quantum beats are observed in the time-resolved correlation measurements^38^.

We measured the ID between consecutive photons only. Past measurements performed on similar InAs/GaAs QD samples have shown that the ID does not degrade much over time differences of about 1.5 μs (refs. ^39,40^). To fuse various sections from the one-dimensional cluster to form higher-dimensionality clusters, the ID should remain high for times comparable with the product of the entanglement length and the photon generation period (about 20–30 ns, only). This value is much shorter than the expected, characteristic decay time of the ID in our QD sample.

**Fig. 3:**
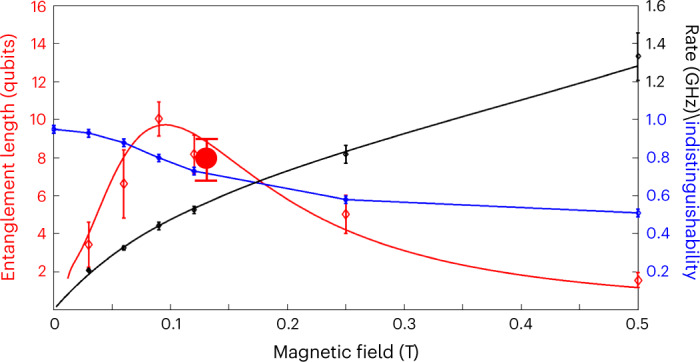
Cluster characterization. The localizable entanglement characteristic length, photon ID and generation rate of the cluster state as a function of the magnetic field. Error bars represent statistical uncertainty of 1 s.d. and standard error propagation calculations. The localizable entanglement and the ID are deduced from the measurements described in Figs. 1 and 2, respectively. The error bars in the photon generation rates result mainly from the experimental uncertainty in the hole’s *g*-factor. The lines represent calculations in which the process map was obtained from our state-evolution model. The red circle represents the localizable entanglement length at *B* = 0.12 T, at which the full process map was measured. See the Supplementary Information for more details.

Figure 3 summarizes the ID properties of the cluster photons, the robustness of the generated entanglement and the photon generation rate as a function of the applied magnetic field.

The entanglement robustness is quantified using localizable entanglement (LE)^41^, defined as the magnitude of the entanglement between two qubits in the cluster after all of the other qubits are projected onto suitable bases. The localizable entanglement decays exponentially with the distance between the two qubits^25,41^. Thus *ζ*_LE_, defined as the characteristic decay length of the localizable entanglement, is a figure of merit characterizing the robustness of the entanglement in the cluster. Our device continuously generates a string of entangled photons. One can select various lengths of consecutive photons from this string by projecting the first and last photons onto the *z*-polarization basis, thereby disentangling the hole spin from the photonic string. All of the photons in the strings are entangled, of course, but the localizable entanglement in the string decays exponentially with a characteristic decay length of *ζ*_LE_ photons. Figure 3 presents *ζ*_LE_ versus the magnetic field strength, where *ζ*_LE_ is deduced from the ratio between two of the measured witnesses (red diamonds) according to:

1 $${\zeta }_{\mathrm{LE}}=-1/\ln \left({\hat{w}}_{3}/{\hat{w}}_{1}\right).$$

The difference between these two witnesses is simply the *D*_cp_ loss due to the application of a single cycle of the protocol. To strengthen our findings, we fully measure the process map of a single cycle of the protocol for a field strength of 0.12 T. The measured map has fidelity of 0.90 ± 0.01 to the ideal process map (see Supplementary Information). The fact that the entire protocol is made of a periodic repetition of exactly the same cycle^23^ enables us to model multiple applications of the measured process map on the spin and thereby calculate the negativity between two qubits in the string as a function of their mutual distance (localizable entanglement). The negativity decays with a characteristic decay length *ζ*_LE_ of about 8 ± 1 (red circle in Fig. 3). To complete our analysis, the measured *ζ*_LE_ versus magnetic field is compared also with our state-evolution model (solid red line), as described in the Supplementary Information and ref. ^36^.

Figure 3 clearly shows an optimum for the entanglement robustness at *B*_*x*_ = 0.09 T, in which *ζ*_LE_ ≃ 10. For stronger fields, the precession period becomes shorter. As a result, the ratio between the radiative and precession times becomes larger, increasing the deviation of the two-qubit gate from the ideal CNOT gate. For weaker fields, the time difference between consecutive pulses increases to a level in which the HH spin dephasing becomes notable.

To complete the device characterization, Fig. 3 also presents the measured ID (in blue) and the cluster generation rate (in black) versus the magnetic field strength. The ID monotonically decreases from 95% as the field increases. This decrease is due to the onset of quantum beats^38^ caused by the Zeeman splitting of the optical transition.

The time between consecutive laser pulses is set to approximately match one-quarter of a precession period. The photon generation rate therefore monotonically increases as a function of the magnetic field. At the optimum point, the generation rate is about 0.5 GHz. The cluster photon generation is deterministic. This means that every laser π-pulse results in the QD spin excitation and emission of an entangled photon. The photon detection, however, is not deterministic as not every emitted photon is detected. The overall detection efficiency of our system is currently better than 1%. The main losses are due to limited light harvesting efficiency from the planar microcavity containing the QD (~20%), free space into fibre coupling (~50%), detector efficiency (~80%) and the overall optical-elements transmission efficiency (~15–20%). After detecting a photon, the detectors have about 25 ns dead-time, in which they cannot detect another photon. We use multiple detectors to detect sequential photons that the device emits about every 2 ns. This further reduces the detection efficiency of multiphoton events. The total single photon detection rate is about 5 MHz with the current system efficiency. The detection rate of four-consecutive photon events is a few hertz only. This detection rate is more than enough to demonstrate and characterize our cluster-state-generating device which, due to the repetitive nature of its production, requires detection of only two and three consecutive photon events. We emphasize here that the non-deterministic detection does not affect the length of the photon string that we generate. It only makes the probability to detect a sequentially larger number of photons exponentially lower. There is an obvious need to improve both the device brightness and the collection efficiency of the optical system so that the generated cluster can be exploited in practical protocols. A clear way to increase the brightness is to embed the QD into a three-dimensional photonic microcavity. This can be achieved either by nanofabrication techniques^21,42^ or by including a tunable, open cavity^40^ in the optical set-up. Both ways are expected to maintain the determinism of the generated cluster while increasing the brightness of the device fourfold, making it close to a deterministic source. At the same time, a better mode matching to the single-mode fibre should also increase the optical system efficiency. Feasible increasing of the overall system efficiency to 50%^40^ will result in a four-photon detection rate of a few tens of megahertz, enabling 25-photon correlation measurement rates of a few hertz. At the same time, a three-dimensional microcavity can considerably shorten the radiative lifetime due to the Purcell effect^21,40,42^. This, in turn, will considerably improve all three cluster states’ figures of merit; the entanglement quality, the ID and the generation rate. The entanglement quality improves by reducing the ratio between the radiative time and the precessions of the spin qubit. The ID improves by broadening the radiative spectral width, thereby reducing the distinguishability due to the Zeeman splitting induced quantum beats. The generation rate increases since the sweet point moves to larger fields. A feasible Purcell factor of ten, for example, is expected to increase the entanglement decay length to about 55 consecutive photons at the sweet point, the ID to better than 90% (limited by the excitation temporal jitter) and the photon generation rate to above 2 GHz (detection rate of single photons to more than 1 GHz).

In summary, we demonstrate a gigahertz-rate deterministic and continuous generation of strings of indistinguishable photons entangled in a cluster state. Our device uses a QD-confined HH spin as the photon entangler, and is optimized by tuning the externally applied magnetic field to achieve a characteristic entanglement decay length of about ten photons, and a photon ID of about 80%. A feasible enhancement of the radiative rate by one order of magnitude is expected to boost the entanglement length to about 55, the photons’ ID to more than 90% and the photon generation rate at these conditions to 2 GHz. The current values may already be used for efficient fusion of cluster states to generate more complicated graph states for demonstrating all-photonic quantum repeaters. Demonstrating this functionality requires better light harvesting efficiencies, as recently demonstrated elsewhere. Our work therefore provides an essential building block for future large-scale quantum communication and information processing.

## Methods

### The sample and optical system

At the heart of our device is an InGaAs self-assembled semiconductor QD, as illustrated in Fig. 1b. The QD contains a confined HH spin qubit^26,30,31,34^. We define the QD’s shortest dimension (~3 nm), the growth direction and the spin quantization axis as the *z*-axis. The externally applied magnetic field direction is defined as the *x*-axis. The QD is embedded in a planar microcavity, formed by two Bragg-reflecting mirrors, facilitating a light harvesting efficiency of about 20% by a 0.85 numerical aperture objective above the QD.

**Extended Data Fig. 1:**
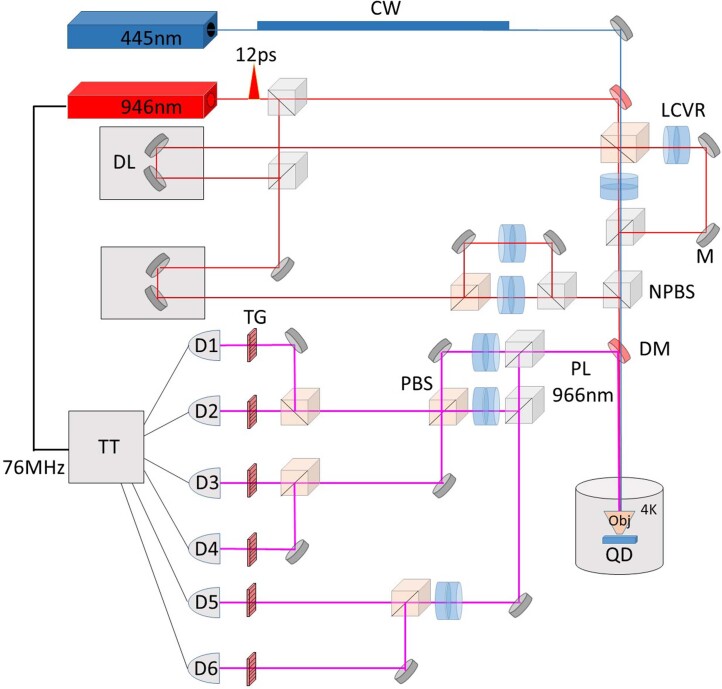
Schematics of the experimental system. CW, continuous wave; DL, Delay line; M, mirror; DM, dichroic mirror; PBS, (NPBS) polarizing (non-polarizing) beam splitter; TG, transmission grating; OBJ, microscope objective; LCVR, liquid crystal variable retarder; Dn, single-photon detector n; TT, Time tagging correlation module.

The experimental system is schematically described in Extended Data Fig. 1. The pulsed laser light is resonantly tuned to the HH-excited trion optical transition^36^, and its power is set to a *π*-area intensity. The polarization of the light is computer-controlled using pairs of LCVRs and PBSs. Very weak, above-bandgap continuous-wave light is also used to stabilize the charge state of the QD^26^. Similar sets of LCVRs and PBSs are used to project the polarization of the collected photons on six different polarization bases. We use highly efficient transmission gratings to spectrally filter the emitted photons. The filtered photons are detected using efficient (>80%) single-photon superconducting detectors, with temporal resolution of about 30 ps. The photons’ detection times are recorded using a time tagging correlation module.

## Online content

Any methods, additional references, Nature Portfolio reporting summaries, source data, extended data, supplementary information, acknowledgements, peer review information; details of author contributions and competing interests; and statements of data and code availability are available at 10.1038/s41566-022-01152-2.

## Supplementary information

Supplementary Information Supplementary Figs. 1–5 and Discussion.

## References

[1] Aspect A, Grangier P, Roger G (1982). Experimental realization of Einstein–Podolsky–Rosen–Bohm *Gedankenexperiment*: a new violation of Bell’s inequalities. Phys. Rev. Lett..

[2] Wehner S, Elkouss D, Hanson R (2018). Quantum internet: a vision for the road ahead. Science.

[3] Pan J-W, Bouwmeester D, Weinfurter H, Zeilinger A (1998). Experimental entanglement swapping: entangling photons that never interacted. Phys. Rev. Lett..

[4] Hong CK, Ou ZY, Mandel L (1987). Measurement of subpicosecond time intervals between two photons by interference. Phys. Rev. Lett..

[5] Briegel H-J, Dür W, Cirac JI, Zoller P (1998). Quantum repeaters: the role of imperfect local operations in quantum communication. Phys. Rev. Lett..

[6] Duan L-M, Lukin MD, Cirac JI, Zoller P (2001). Long-distance quantum communication with atomic ensembles and linear optics. Nature.

[7] Raussendorf R, Briegel HJ (2001). A one-way quantum computer. Phys. Rev. Lett..

[8] Rudolph T (2017). Why I am optimistic about the silicon-photonic route to quantum computing. APL Photon.

[9] Zwerger M, Dür W, Briegel HJ (2012). Measurement-based quantum repeaters. Phys. Rev. A.

[10] Briegel HJ, Raussendorf R (2001). Persistent entanglement in arrays of interacting particles. Phys. Rev. Lett..

[11] Hein M, Eisert J, Briegel HJ (2004). Multiparty entanglement in graph states. Phys. Rev. A.

[12] Buterakos D, Barnes E, Economou SE (2017). Deterministic generation of all-photonic quantum repeaters from solid-state emitters. Phys. Rev. X.

[13] Pichler H, Choi S, Zoller P, Lukin MD (2017). Universal photonic quantum computation via time-delayed feedback. Proc. Natl Acad. Sci. USA.

[14] Azuma K, Tamaki K, Lo H-K (2015). All-photonic quantum repeaters. Nat. Commun..

[15] Munro WJ, Stephens AM, Devitt SJ, Harrison KA, Nemoto K (2012). Quantum communication without the necessity of quantum memories. Nat. Photon..

[16] Larsen MV, Guo X, Breum CR, Neergaard-Nielsen JS, Andersen UL (2019). Deterministic generation of a two-dimensional cluster state. Science.

[17] Li J-P (2020). Multiphoton graph states from a solid-state single-photon source. ACS Photon..

[18] Istrati D (2020). Sequential generation of linear cluster states from a single photon emitter. Nat. Commun..

[19] Besse J-C (2020). Realizing a deterministic source of multipartite-entangled photonic qubits. Nat. Commun..

[20] Akopian N (2006). Entangled photon pairs from semiconductor quantum dots. Phys. Rev. Lett..

[21] Senellart P, Solomon G, White A (2017). High-performance semiconductor quantum-dot single-photon sources. Nat. Nanotechnol..

[22] Appel MH (2021). Coherent spin-photon interface with waveguide induced cycling transitions. Phys. Rev. Lett..

[23] Lindner NH, Rudolph T (2009). Proposal for pulsed on-demand sources of photonic cluster state strings. Phys. Rev. Lett..

[24] Berezovsky J, Mikkelsen MH, Stoltz NG, Coldren LA, Awschalom DD (2008). Picosecond coherent optical manipulation of a single electron spin in a quantum dot. Science.

[25] Schwartz I (2016). Deterministic generation of a cluster state of entangled photons. Science.

[26] Cogan D (2018). Depolarization of electronic spin qubits confined in semiconductor quantum dots. Phys. Rev. X.

[27] Bechtold A (2015). Three-stage decoherence dynamics of an electron spin qubit in an optically active quantum dot. Nat. Phys..

[28] Kiraz A, Atatüre M, Imamoğlu A (2004). Quantum-dot single-photon sources: prospects for applications in linear optics quantum-information processing. Phys. Rev. A.

[29] Schwartz I (2015). Deterministic writing and control of the dark exciton spin using single short optical pulses. Phys. Rev. X.

[30] Gerardot BD (2008). Optical pumping of a single hole spin in a quantum dot. Nature.

[31] Brunner D (2009). A coherent single-hole spin in a semiconductor. Science.

[32] Benny Y (2012). Excitation spectroscopy of single quantum dots at tunable positive, neutral, and negative charge states. Phys. Rev. B.

[33] Benny Y (2014). Electron-hole spin flip-flop in semiconductor quantum dots. Phys. Rev. B.

[34] Loss D, DiVincenzo DP (1998). Quantum computation with quantum dots. Phys. Rev. A.

[35] Cogan D, Peniakov G, Su Z-E, Gershoni D (2020). Complete state tomography of a quantum dot spin qubit. Phys. Rev. B.

[36] Cogan D, Su Z-E, Kenneth O, Gershoni D (2022). Spin purity of the quantum dot confined electron and hole in an external magnetic field. Phys. Rev. B.

[37] Santori C, Fattal D, Vučković J, Solomon GS, Yamamoto Y (2002). Indistinguishable photons from a single-photon device. Nature.

[38] Legero T, Wilk T, Hennrich M, Rempe G, Kuhn A (2004). Quantum beat of two single photons. Phys. Rev. Lett..

[39] Wang H (2016). Near-transform-limited single photons from an efficient solid-state quantum emitter. Phys. Rev. Lett..

[40] Tomm N (2021). A bright and fast source of coherent single photons. Nat. Nanotechnol..

[41] Verstraete F, Popp M, Cirac JI (2004). Entanglement versus correlations in spin systems. Phys. Rev. Lett..

[42] Liu F (2018). High Purcell factor generation of indistinguishable on-chip single photons. Nat. Nanotechnol..

