# Supplementary material for: Deterministic generation of indistinguishable photons in a cluster state
Source: Nat Photonics. 2023 Feb 9;17(4):324–9. doi: 10.1038/s41566-022-01152-2 (PMC10091623; doi:10.1038/s41566-022-01152-2)
Supplement: Supplementary file 1 — Supplementary Figs. 1–5 and Discussion. [file 41566_2022_1152_MOESM1_ESM.pdf]

nature photonics

Article https://doi.org/10.1038/s41566-022-01152-2

Deterministic generation of indistinguishable photons in a cluster state

In the format provided by the authors and unedited

CONTENTS

S1. The state-evolution model 2

S2. Measuring the process map of the repeated cycle of the protocol 4

S3. Comparison of photon indistinguishability using the DE and HH spins as entanglers 9

S4. The optical transitions of the positive trion in the presence of in-plane magnetic field. 10

S5. Deterministic photon generation by a laser $\pi-$pulse tuned to the hole-trion* resonance. 11

S6. Probabilistic cluster fusion 14

References 15

S1. THE STATE-EVOLUTION MODEL

Here we outline the state evolution model, which describes the actual single cycle of the periodic protocol. The cycle is ideally composed of two gates. A CNOT gate between the Quantum-Dot (QD) spin-qubit and the emitted photon polarization-qubit, followed by a Hadamard gate acting on the spin qubit only. The CNOT gate is realized by the pulsed optical excitation of the Heavy-Hole (HH) spin and the resulting single-photon emission. The Hadamard gate is realized by the timed free coherent precession of the HH around the direction of the externally applied magnetic field. We developed the state evolution model in order to realistically describe the process map and its deviation from the ideal map, composed of the ideal gates.

The optical excitation of the HH results in a positively charged trion composed of two paired holes and a single electron (Fig. S2a). The two holes and the single electron spins interact via the hyperfine interaction with nuclear spins in their vicinity and with the externally applied magnetic field. In Ref. [1], we developed a model that quantitatively describes both spins' evolution under these conditions, focusing particularly on the relevant regime, where the external field is comparable in magnitude to one standard deviation of the effective nuclear magnetic field (Overhauser field) due to random statistical fluctuations in the nuclei spins [2].

In Ref. [3], we show that under these conditions the central spin $\vec{S}(t)$ evolution is given by:

$$\vec{S}(t) = \bar{G}\vec{S}_0 \tag{1}$$

where $\vec{S}_0 = \vec{S}(0)$ is the central spin initial value and $\bar{G}$ is a 3×3 tensor whose elements can be calculated numerically as explained in Ref [3]. We mark by $\bar{G}^{HH}$ and $\bar{G}^{trion}$ the tensors for the HH and trion.

Each cycle starts with an initial HH spin state $\vec{S}_0$, which is the state in which the HH was left at the end of the previous cycle. The photons emitted by the QD during previous cycles can be safely disregarded.

A 12ps π-area laser pulse converts the HH state to an excited positive trion state. The optical transitions between these two spin qubits are governed by the following Π -system optical polarization selection rules [4]:

$$\begin{aligned} &|\Uparrow\rangle \xleftrightarrow{|-Z\rangle} |\Uparrow\Downarrow\uparrow\rangle\,, \\ &|\Downarrow\rangle \xleftrightarrow{|+Z\rangle} |\Downarrow\Uparrow\downarrow\rangle\,. \end{aligned} \tag{2}$$

where $|+Z\rangle$ and $|-Z\rangle$ are photons with right and left circular polarizations or angular momentum of +1 and -1, respectively along the z-direction. Since the short laser pulse and the immediate optical phonon assisted non-radiative relaxation of the trion to its ground state can be considered instantaneous it follows that a short pulse polarized $|+X\rangle = (|+Z\rangle + |-Z\rangle)/\sqrt{2}$, coherently converts the HH spin state into a similar trion state[3, 5] described by $\vec{S}_0$. The trion then radiatively decays exponentially ($\sim e^{-t/\tau_{photon}}$ with $\tau_{photon} = 400\ ps$) into an entangled HH+photon state.

We proceed by separating the temporal evolution into three domains: the trion evolution before the emission, the emission itself and the HH evolution after the emission. The evolution during the first and third domains is described by Eq. 1. In the following we discuss the second part which includes both qubits: the emitted photon and the spin.

Note that while above we used $\vec{S}$ to describe the QD spin, an equivalent description in terms of a density matrix is

$$\hat{\rho}_{2\times2} = \frac{1}{2}\left(\hat{\sigma}_0 + S_x\hat{\sigma}_x + S_y\hat{\sigma}_y + S_z\hat{\sigma}_z\right) \tag{3}$$

with $\hat{\sigma}_i$ being the Pauli matrices and $\hat{\sigma}_0$ the identity matrix. According to the polarization selection rules for the optical transition between the trion and the HH spins, described by Eq. 2, a photon emission occurring right after the moment $t = t'_+$, results in the spin+photon state

$$\hat{\rho}_{4\times4}(t'_+) = \begin{bmatrix} 0 & & \\ & \hat{\rho}_{2\times2}(t'_-) & \\ & & 0 \end{bmatrix}. \tag{4}$$

Projecting this on the photon state $\frac{1}{2}(I + \vec{m}\cdot\vec{\sigma})$, where $\vec{m}$ is the photon's normalized Stokes vector, we obtain after tracing over the photon, the HH state

$$\frac{1}{2}\begin{bmatrix} (1+S_z)(1-m_3) & (S_x - iS_y)(m_1 - im_2) \\ (S_x + iS_y)(m_1 + im_2) & (1-S_z)(1+m_3) \end{bmatrix}. \tag{5}$$

where $\vec{S} = \vec{S}^{trion}(t'_-)$ right before the photon emission. The (trace) normalization of this gives the probability $p = \frac{1}{2}(1 - S_z m_3)$ to find the photon $\hat{m}$-polarized and the expectation value of $\vec{\sigma}$ gives the HH spin

$$p\vec{S}^{HH}(t'_+) = \frac{1}{2}(m_1S_x - m_2S_y, m_1S_y + m_2S_x, S_z - m_3) \tag{6}$$

We may write this as $p\vec{S}^{HH}(t'_+) \equiv \bar{T}^m\vec{S}^{trion}(t'_-)$ where $\bar{T}^m$ is defined by Eq. 6. Note that $\bar{T}^m$ is an affine rather than a linear transformation.

Collecting everything together, this suggests that the spin at the end of the cycle (occurring at the moment t_{pulse} of the next pulse) should satisfy

$$P^{(m)} \left\langle \overrightarrow{S}(t_{pulse}) \right\rangle = \frac{1}{\tau_{photon}}(1 - e^{-\frac{t_{pulse}}{\tau_{photon}}}) \int d\mathcal{P} \int_0^{t_{pulse}} e^{-\frac{t'}{\tau_{photon}}} dt' \bar{G}^{HH}(t_{pulse} - t') \bar{T}^m \bar{G}^{trion}(t') \vec{S}_0 b \tag{7}$$

where

$$d\mathcal{P} = \frac{1}{(2\pi)^{3/2}} \exp\left(-\frac{1}{2}B^2\right) d^3B \tag{8}$$

is the probability element of the normalized nuclear field [3], and $P^{(m)}$ is the probability to find the emitted photon $\hat{m}$-polarized. $P^{(m)}$ may be obtained from

$$1 - 2P^{(m)} = \frac{m_3}{\tau_{photon}}(1 - e^{-\frac{t_{pulse}}{\tau_{photon}}}) \int d\mathcal{P} \int_0^{t_{pulse}} e^{-\frac{t'}{\tau_{photon}}} dt' \left(\hat{z} \cdot \bar{G}^{trion}(t') \vec{S}_0\right). \tag{9}$$

In practice $e^{-\frac{t_{pulse}}{\tau_{photon}}} \ll 1$ may be safely neglected.

We solve the integrals in Eq. 7,9 numerically [6] with the QD carriers' g-factor and hyperfine tensors as measured independently and described elsewhere [3].

We use this model to calculate the expected values of the four cluster-state witnesses displayed in Fig. 1g of the main article. Similarly, we use the model for calculating the expected process map [7] and the localizable entanglement characteristic length [8, 9] as a function of the externally applied magnetic field, as displayed in Fig. 3 of the main article. As can be seen in both figures the model quite accurately describes the measurements.

S2. MEASURING THE PROCESS MAP OF THE REPEATED CYCLE OF THE PROTOCOL

As discussed above, the cluster state is generated by repeatedly applying the same process on the QD confined spin. This means that one can fully characterize the cluster quantum state for any number of qubits if the process-map is accurately known [7, 9]. The process map, which is schematically described in Fig. S1a, maps the spin qubit state into an entangled spin-photon two qubit state. Since the spin quantum state can be fully described by a 2x2 density matrix and the spin+photon state by a 4x4 density matrix, it follows that the process map can be fully described

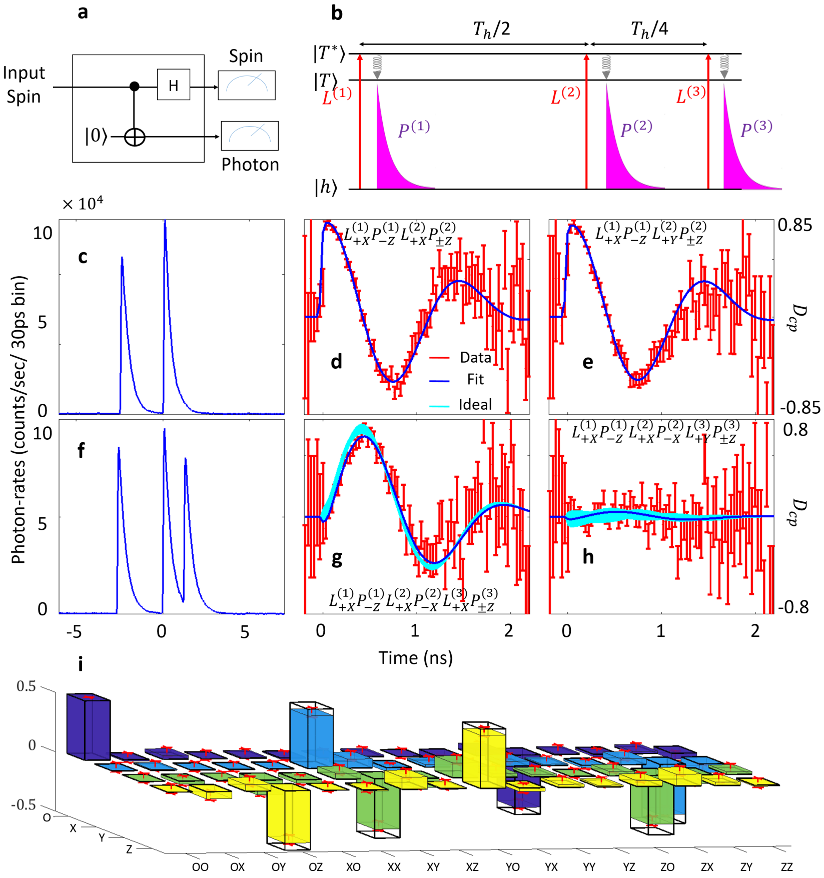

Figure S1. Measurement of the process map for an applied magnetic field of 0.12T. **a,** Schematics description of the process map acting on the HH spin. **b,** Schematic description of the pulse sequence and resulted photon emission used for measuring the process map elements for initialized HH spin $|\pm Z\rangle$ states. $L_i^{(n)}$ ($P_i^{(n)}$) mark the n's laser pulse (single-photon detected) where i marks the polarization. **c, d-e,** Time resolved emission and degree-of-circular-polarizations (D_{cp}) measurements for characterizing the HH $|+Z\rangle$ input spin state. **f, g-h,** Time resolved emission and D_{cp} measurements for characterizing the spin+photon state, resulting from application of the process map on the $|+Z\rangle$ input spin state. The error bars represent statistical one standard deviation uncertainties deduced from a temporally integrated total number of about 15000 2- and 10000 3-sequential photons detection events in **d-e** and **g-h**, respectively. The blue lines represent the best fitted model results [5]. The light-blue lines represent the expected results assuming ideal process map. **i,** The measured (filled colored bars) and ideal (empty bars) process map. X, Y, Z, and O stands for the corresponding Pauli and identity matrices basis representation, respectively.

by a 4x16 positive and trace-preserving matrix which contains 64 real matrix elements, as presented in Fig. S1a.

In Ref. [7] we describe a novel way for obtaining the process map from a set of time resolved polarization sensitive two and three photon correlation measurements. In Fig. S1b. we schematically describe the experimental method where we used a 2-pulse-experiment to initialize a HH spin qubit and then to measure the resulting HH quantum state [5]. Similarly, a 3-pulse-experiment is used to initialize the HH spin, apply one cycle of the process on the initialized spin and then to measure the resulted entangled spin+photon state.

In Fig. S1c we present time-resolved two-pulse-correlation-measurements in which the input HH was initialized to $|+Z\rangle$ state and then its state was measured [5]. Fig. S1d and e show the measured time resolved degree of circular polarization ($D_{cp}(t)$) of the emission resulting from a second pulse polarized +X and +Y respectively.

Similarly, we initialize the HH-state to six different states from three orthogonal bases [7, 10]. For each of those 6 states, we used 2 $D_{cp}(t)$ two-photon correlation measurements (not shown), like the measurements displayed in Fig. S1d and Fig. S1e for tomography.

Fig. S1f shows time-resolved three-pulse-correlation-measurement where one cycle of the process is applied to the $|+Z\rangle$ input state and the resulting spin+photon state is measured. In this experiment, the second photon is projected on the -X polarization. Fig. S1g and Fig. S1h show the time resolved $D_{cp}(t)$ of the three-photon correlations measurements resulting from a third pulse polarized +X and +Y, respectively [5].

Fig. S1i shows the results of the full-tomographic measurements of the process map. For acquiring these measurements, for the 6 HH spin initializations, we applied one cycle of the protocol and measured the resulting spin+photon state by projecting both the spin and the photon on different orthogonal polarization bases [11]. For characterizing each of these 6 spin+photon states, we used 12 $D_{cp}(t)$ 3-photon correlations measurements like the ones presented in Fig. S1g and Fig. S1h.

Lastly, in order to obtain the physical process map that best fits our measured results, we use a novel minimization method [7]. In Fig. S1i we compare between the obtained process-map and the ideal one. The fidelity [9, 12] of the measured map to the ideal one is 0.9.

We quantify the entanglement robustness in the generated cluster by the notion of localizable entanglement (LE) [8]. LE is defined as the entanglement left in a two-qubit state after the other qubits in the cluster were projected on appropriate polarization bases.

Since our device generates continuously long string of entangled photons, one can select various sections of consecutive photons from this string by projecting the first and last photons on the z-

polarization basis, disentangling the hole spin from the infinite string. The localizable entanglement decays exponentially within the section that one cuts this way according to

$$N(d) = N_{nn} \exp\left(-(d-1)/\zeta_{LE}\right), \tag{10}$$

where N_{nn} is the negativity between nearest-neighbor qubits, d is the distance between the qubits, and ζ_{LE} is the characteristic decay-length of the LE. Figure S2 shows the LE between two qubits in the cluster as a function of the qubit distance in the string. To quantify the LE decay, we utilize the fact that the cluster generation protocol is made of repeating identical cycles, so if a single cycle is fully characterized by finding its process map, an arbitrary n-photon long cluster state can be reconstructed by applying the fully characterized process map n-times. We note that this approach rests on the assumption that the noise in the system is Markovian. Previous studies of electronic spins in semiconductors (including ours [1, 4] suggest a non-Markovian decay in the spin coherence due to coupling to a nuclear spin bath. For the short times that we are interested in (20-30 ns), and for the hole spin, which is coupled only weakly to the nuclear spin, the Markovian assumption is adequate (see the original proposal of Lindner and Rudolph [13] and later Ref. [14] where this point is studied). The effect of the Markovian approximation, if any, may only lead to a small underestimation of the entanglement robustness [14].

For finding the LE as a function of qubit distance in the cluster, we then project all the photons in the constructed state, apart from the two qubits in question, on an appropriately chosen polarization bases. Then we quantify the entanglement left in the 2-qubit state using the Peres criterion, which gives a Negativity of 0.5 for maximally entangled Bell states. We present the LE results in Fig. S2 as a function of the distance between two qubits for six different magnetic fields. The shaded red area represents one standard deviation of the measured LE decay for 0.12T, generated by repeatedly applying the measured process map. The colored solid lines are acquired from our model, described in Sec. S1, with model parameters as independently measured elsewhere [1]. We add to Fig. S2 the measured witnesses $\hat{w}_1 = P_Z^{(n-1)} S_Y$, $\hat{w}_3 = P_Z^{(n-1)} P_X^{(n)} S_Z$ and $\hat{w}_4 = P_Z^{(n-1)} P_X^{(n)} P_Z^{(n+1)}$. Where $\hat{w}_1$ represents the spin initialization as a function of the magnetic field, $\hat{w}_3$ represents a spin-photon state after application of one cycle of the protocol on the initialized spin, and $\hat{w}_4$ is the cluster state generator. We normalize the LE decay to the spin initialization $\hat{w}_1$. It is apparent that the model calculated LE decay concur with the results of the measured witnesses and the LE decay deduced from the measured process map. We calculate the characteristic decay length of ζ_{LE} photons by either fitting the LE decay with Eq. 10 or from the ratios of $\hat{w}_3$ and $\hat{w}_1$ according to Eq. 1 in the main manuscript. ζ_{LE} as a function of the magnetic field is presented in Fig. 3 in

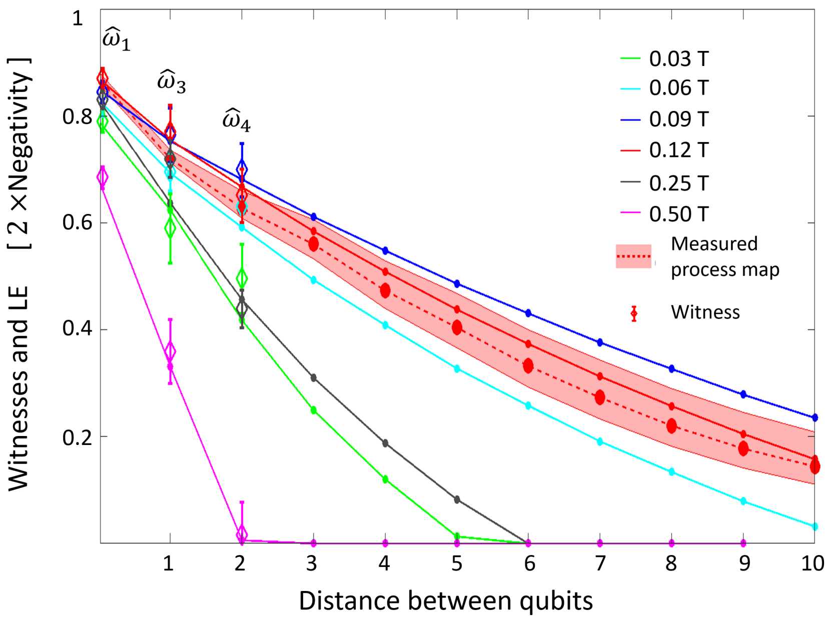

Figure S2. The Localizable entanglement (LE) between two qubits in the cluster, as a function of the distance between these qubits. The LE is the entanglement between these two-qubits (using Peres criterion -negativity) after projecting all the other qubits in the cluster on the appropriate (linearly polarized) bases. We normalize the LE decay to the measured spin initialization measurement ($\hat{w}_1$ witness). The colored lines represent the LE decay for various magnetic fields, calculated using the model in sec. S1. The dotted red line represents the LE decay, deduced by repeatedly applying the measured process map at 0.12T. The shaded red area represents the uncertainty in the LE decay due to one standard deviation experimental uncertainty in the measured process map. The measured witnesses and their error bars are defined in Fig. 1g in the main text.

the main article.

The LE better characterizes the robustness of the entanglement in the cluster than the more commonly used fidelity for a given specific multi photon cluster state, which can be also deduced from the process map. For comparison, the fidelity of three entangled photon state to the ideal one as deduced from the measured process map at 0.12 T is 0.78 ± 0.01. The fidelity of the three (eight) photon state at the sweet point of the magnetic field (0.09 T) is 0.81 ± 0.01 (0.53 ± 0.01).

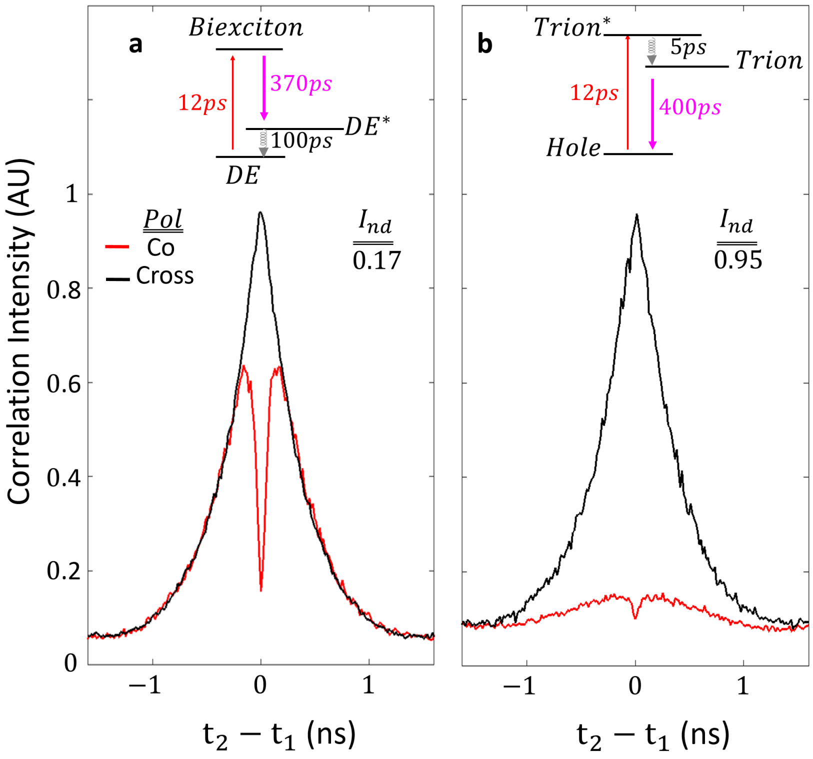

Figure S3. Comparison between the photon indistinguishabilities resulting from using the DE or the HH as entanglers. a, (b,) Indistinguishability measurements between two consecutive photons from a cluster where the DE (HH) is the entangler. The solid lines represent the measured second-order correlation function $g^{(2)}(t_2 - t_1)$. For these measurements the excitation was rectilinearly polarized horizontally, while the correlation measurements represent an average over horizontal and vertical polarization measurements. The measured indistinguishability is defined as $I_{nd} = 1 - A_{co}/A_{cross}$, where $A_{co}(A_{cross})$ is the area under the co-(cross-) polarized correlation function, represented by the red (black) solid line. The areas are calculated after subtracting the unpolarized background. The inset in **a** (**b**) describes the energy levels and the transition rates between the levels for the DE (HH).

S3. COMPARISON OF PHOTON INDISTINGUISHABILITY USING THE DE AND HH SPINS AS ENTANGLERS

We compare the photon-indistinguishability resulting from using the dark exciton (DE) as entangler (Fig. S3a) with that of the HH (Fig. 2b in the main article and Fig. S3b) without applying an external magnetic field. We use the HOM setup [15, 16] illustrated in Fig. 2a of the

main article. As explained in the main article, we calculate the indistinguishability from the ratio between the second-order interference of co- to cross-polarized photons. The DE-cluster photons indistinguishability amounts to 17%, much lower than that of the HH-cluster, which amounts to 95%.

The indistinguishability between photons emitted from a quantum source of single-photons depends on the initial and final states' temporal stability. The indistinguishability can be roughly estimated by [17, 18]:

$$I_{nd} = [(1 + \tau_{photon}/\tau_{final})(1 + \tau_{init}/\tau_{photon})]^{-1} \tag{11}$$

In Eq. 11, τ_{init} is the initial state's generation-time ("jitter"), τ_{final} is the final state's lifetime, and τ_{photon} is the photon'radiative lifetime. To achieve high indistinguishability, $\tau_{init}/\tau_{photon}$ should be minimized while $\tau_{final}/\tau_{photon}$ should be maximized.

The insets to Fig. S3a and Fig. S3b illustrate the energy level structures and the relevant transition times between these levels for the DE and for the HH, respectively. Using these rates in Eq. 11 results in indistinguishability of about 20% for the DE-cluster and 95% for the HH-Trion photons. These estimates agree with the experimental results of 17% and 95% for the DE and HH, respectively.

S4. THE OPTICAL TRANSITIONS OF THE POSITIVE TRION IN THE PRESENCE OF IN-PLANE MAGNETIC FIELD.

The externally applied magnetic field reduces the indistinguishability of the HH-cluster photons. In Fig. S4a, we schematically describe the HH and positive-trion energy levels and the selection rules for optical transitions between these levels in the presence of an in-plane magnetic field. The field lifts the degeneracy of both the HH and the trion qubits. The resulted HH precession is used for implementing the Hadamard gate on the spin. The precession of the trion is used for tomography of the spin qubit [5]. In Fig. S4b-d, we show polarization-sensitive spectral measurements of the X^{+1} spectral line for various magnetic fields. Without a magnetic field, the line is completely degenerate. For a small field of 0.09 Tesla, the field induced degeneracy removal results in a small broadening of the spectral line. This degeneracy removal is more than enough, however, for our purpose, as shown in the main text. For a field of 1.00 Tesla, the spectral line is split into four components, rectilinearly cross-polarized, in perfect agreement with the scheme of Fig. S4a.

Fig. 2b in the main article presents indistinguishability measurements of the HH-cluster photons for various magnetic fields. As the field increases, the average (over both rectilinear polarizations)

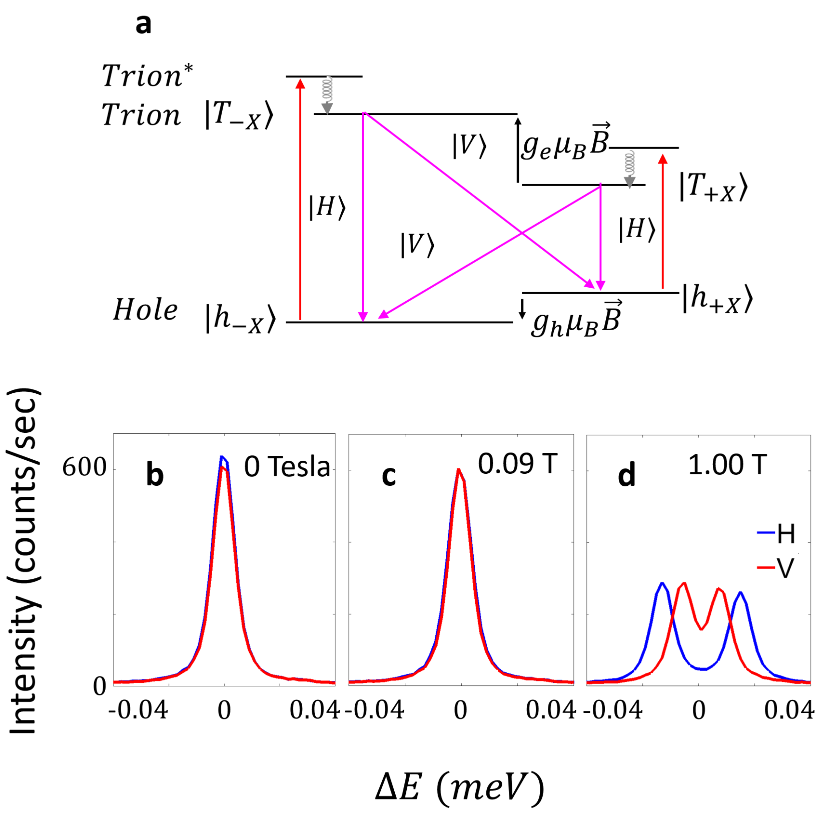

Figure S4. Spectroscopy of the positive trion (X^{+1}) in the presence of externally applied in-plane magnetic field. a, Schematics of the Trion-HH energy levels and optical transitions in the presence of an in-plane external magnetic field. Here H=X (V=Y) describes horizontally (vertically) rectilinearly polarized optical transition. **b-d,** Polarization-sensitive PL spectral measurements of the Trion-HH transition for various magnetic-fields.

indistinguishability decreases due to the field-induced spectral broadening. At large fields, when the Zeeman splitting surpasses the radiative linewidth, quantum beats are observed in the time-dependent correlation measurements [19]. The quantum beats oscillation frequency exactly matches the energy difference between the V-polarized components of the X^{+1} line in Fig. S4d.

S5. DETERMINISTIC PHOTON GENERATION BY A LASER π−PULSE TUNED TO THE HOLE-TRION* RESONANCE.

We show that the cluster state photons are generated deterministically, despite the fact that the laser's temporal pulse width is comparable to the lifetime of the resonantly excited state. the physical process is described in the inset to Fig. S5a, which schematically describes the energy levels

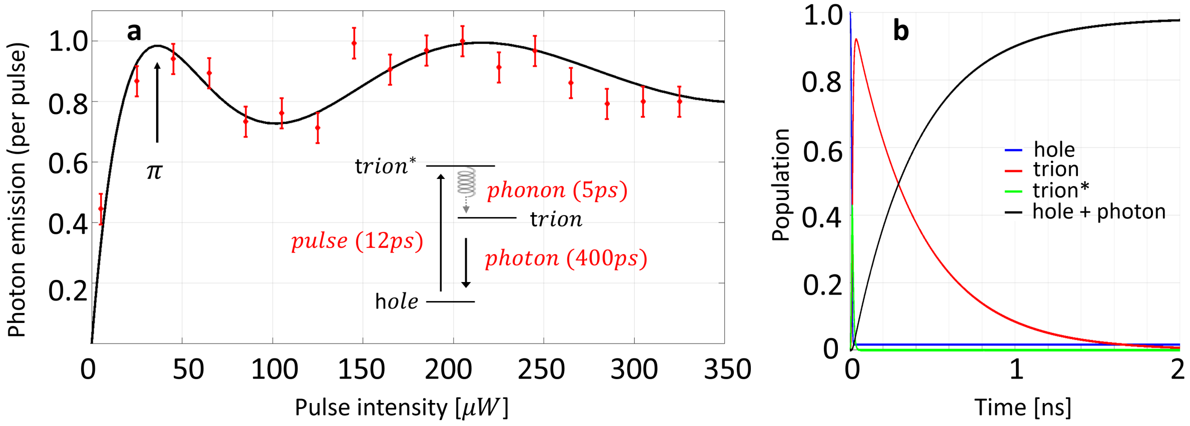

Figure S5. Deterministic cluster photons generation. a, The normalized X^{+1} (trion-hole optical transition) emission intensity as a function of the quasi-resonant pulse excitation power into the hole-trion* absorption resonance. A vertical black arrow marks the power at which a π-pulse is obtained. The red error bars represent one standard deviation of the experimental uncertainty using 500000 total number of single photon detection events. The black solid line represents the $|hole + photon\rangle$ population after the pulse as calculated by a Lindblad model fit using Eq. 12. The inset describes the 3-level system and the transition times between these levels used in the model. **b,** The model calculated populations of the 4 different QD-states as a function of time after a π-area pulse is applied. The $|hole + photon\rangle$ population reaches 98%. The deviation from 100% may result from a slight detuning of the pulse energy from the hole-trion* absorption resonance.

involved and the transition rates between these levels. A 12 ps long pulse converts the hole to the excited trion* state, which rapidly decays non-radiatively to the ground state trion via a short few ps long optical-phonon-assisted transition [20]. The trion, in turn, radiatively decays back to the HH state by emitting a photon. To show that the photon generation is indeed deterministic, we perform a measurement where we vary the intensity of the exciting resonant laser pulse tuned to the hole-trion* absorption resonance while detecting the photons emitted from the trion-hole optical transition. The measured results, displayed in Fig. S5a, are perfectly explained by our model calculations as presented by the solid line in Fig. S5a. Indeed, since the excitation and emission transitions are connected via a fast non-radiative transition comparable in time to the laser pulse width the population which decays during the pulse does not reach the ground HH state and the population transfer to the trion state at the end of the pulse is almost deterministic.

We use a Lindblad time evolution model, described by [21]:

$$\dot{\rho} = -\frac{i}{\hbar}[H, \rho] + \sum_k D(C_k)\rho \tag{12}$$

with H being the system's Hamiltonian, [...] is the commutator, and the summation is over the relevant decay mechanisms C_k where:

$$D(C)\rho = C\rho C^\dagger - \frac{1}{2}(C^\dagger C\rho + \rho C^\dagger C) \tag{13}$$

is the Lindblad super-operator. In a simplified 4-states system, the Hamiltonian describing such a pulse in the $|hole\rangle$, $|trion*\rangle$, $|trion\rangle$, $|hole + photon\rangle$ basis is:

$$H = \begin{bmatrix} \Delta & \Omega(t) & 0 & 0 \\ \Omega(t) & 0 & 0 & 0 \\ 0 & 0 & 0 & 0 \\ 0 & 0 & 0 & 0 \end{bmatrix}. \tag{14}$$

The parameter $\Omega(t)$ describes the temporal evolution of the pulse such that:

$$\Omega(t) = \frac{\Theta}{\sqrt{8\pi\sigma_p^2}} \exp\left(-\frac{t^2}{2\sigma_p^2}\right) \tag{15}$$

where σ_p is the pulse temporal width and Θ is the pulse area (π for a full population transfer). We add to the Hamiltonian a small pulse detuning from the hole-trion* absorption resonance, marked by Δ. Ideally, $\Delta = 0$, but in the experiments, a small detuning relative to the relatively large spectral width of the resonance (about $300\mu eV$ [20]), is possible. The decay mechanisms in Eq. 12 are described by $C = \sqrt{1/\tau}\hat{a}$, where τ is the exponential decay times of the phonon (about 5ps) or photon (400ps), and $\hat{a}$ is a lowering operator between the two relevant levels.

In Fig. S5a, the red error bars describe the normalized measurements, and the solid line describes the Lindblad model with the independently measured rates $\tau_{phonon} = 5ps$, $\tau_{photon} = 400ps$. A small detuning of $\Delta = 20\mu eV$ of the pulse relative to the resonance width (about $300\mu eV$) explains the slight height difference between the first (π) and second (3π) peak. The ratio between the phonon time (5ps) and the pulse width (12ps) determines the visibility of the oscillations. Fig. S5b shows the time-dependent population of the 4 QD-states from the applied π-area intensity pulse. The $|hole + photon\rangle$ population at that intensity reaches 98%, indicating almost complete deterministic generation which can still be improved by better tuning the excitation pulse.

S6. PROBABILISTIC CLUSTER FUSION

The indistinguishability of our cluster photons enables "fusing" two 1D cluster strings into one string, thereby increasing the cluster size and its dimensionality. Since the fusion use Bell state measurements (BSM) between two photons, one from each string, it is probabilistic in nature. Probabilistic fusion was already considered in the original proposal of Lindner and Rudolph [13] and later in Ref. [22]. We propose to use a type II fusion [23], by which a BSM between two photons from two different clusters, if successful, provides an entangling link between the clusters. We will tune the magnetic field to achieve an indistinguishability of above 90% while maintaining an entanglement length of 8 photons in the generated 1D cluster state. With the cluster photons being 90% indistinguishable, the gate fidelity of a successful BSM measurement is about 90% [24], and therefore the robustness of the entanglement of the link between the clusters will be comparable to that of the links within the parent clusters (entanglement decay length of 8 photons) [25]. Assuming that the success probability of the fusion gate is $p = 0.5 \times 0.9 = 0.45$ and considering that the measured two photons in the fusion process are lost in the process, one can see that the probabilistic fusion will increase the cluster in a scalable manner. Let us consider fusing a cluster of size N with a cluster of size M. For the fusion gate to result in a larger cluster, the following restriction on M needs to be fulfilled:

$$p(N + M - 2) + (1 - p)(N - 1) > N, \tag{16}$$

where we consider the fact that an unsuccessful measurement reduces the number of photons in the cluster N by one. The condition of Eq. 16 results in a minimal cluster state length of $M > (p + 1)/p \sim 3.25$. Therefore, since our M=8 photon long cluster is more than twice that length, at least conceptually, this length provides already a route for fast generation of large cluster states in a probabilistic manner.

[1] Cogan, D., Su, Z.-E., Kenneth, O. & Gershoni, D. Spin purity of the quantum dot confined electron and hole in an external magnetic field. *Physical Review B* **105**, L041407 (2022).

[2] Merkulov, I. A., Efros, A. L. & Rosen, M. Electron spin relaxation by nuclei in semiconductor quantum dots. *Physical Review B* **65**, 205309 (2002).

[3] Cogan, D., Su, Z.-E., Kenneth, O. & Gershoni, D. The coherence of quantum dot confined electron- and hole-spin in low external magnetic field. *arXiv:2108.05173* (2021). http://arxiv.org/abs/2108.05173v1.

[4] Cogan, D. *et al.* Depolarization of electronic spin qubits confined in semiconductor quantum dots. *Physical Review X* **8**, 041050 (2018).

[5] Cogan, D., Peniakov, G., Su, Z.-E. & Gershoni, D. Complete state tomography of a quantum dot spin qubit. *Physical Review B* **101**, 035424 (2020).

[6] For certain specific photon polarizations $\hat{m}$, one may show analytically that some components of the integral vanish by reflection symmetries.

[7] Cogan, D., Peniakov, G., Kenneth, O., Don, Y. & Gershoni, D. Quantum tomography of entangled spin-multiphoton states. *Physical Review Applied* **18**, 024055 (2022).

[8] Verstraete, F., Popp, M. & Cirac, J. I. Entanglement versus correlations in spin systems. *Physical Review Letters* **92**, 027901 (2004).

[9] Schwartz, I. *et al.* Deterministic generation of a cluster state of entangled photons. *Science* **354**, 434–437 (2016).

[10] Schwartz, I. *et al.* Deterministic coherent writing of a long-lived semiconductor spin qubit using one ultrafast optical pulse. *Physical Review B* **92**, 201201 (2015).

[11] James, D. F. V., Kwiat, P. G., Munro, W. J. & White, A. G. Measurement of qubits. *Physical Review A* **64**, 052312 (2001).

[12] Jozsa, R. Fidelity for mixed quantum states. *Journal of Modern Optics* **41**, 2315–2323 (1994).

[13] Lindner, N. H. & Rudolph, T. Proposal for pulsed on-demand sources of photonic cluster state strings. *Physical Review Letters* **103**, 113602 (2009).

[14] McCutcheon, D. P. S., Lindner, N. H. & Rudolph, T. Error distributions on large entangled states with non-markovian dynamics. *Physical Review Letters* **113**, 260503 (2014).

[15] Hong, C. K., Ou, Z. Y. & Mandel, L. Measurement of subpicosecond time intervals between two photons by interference. *Physical Review Letters* **59**, 2044–2046 (1987).

[16] Santori, C., Fattal, D., Vučković, J., Solomon, G. S. & Yamamoto, Y. Indistinguishable photons from a single-photon device. *Nature* **419**, 594–597 (2002).

[17] Kiraz, A., Atatüre, M. & Imamoğlu, A. Quantum-dot single-photon sources: Prospects for applications in linear optics quantum-information processing. *Physical Review A* **69**, 032305 (2004).

[18] Gantz, L. *et al.* A new study of on-demand emission of indistinguishable single photons from single quantum dots. In *Conference on Lasers and Electro-Optics* (OSA, 2017).

[19] Legero, T., Wilk, T., Hennrich, M., Rempe, G. & Kuhn, A. Quantum beat of two single photons. *Physical Review Letters* **93**, 070503 (2004).

[20] Benny, Y. *et al.* Excitation spectroscopy of single quantum dots at tunable positive, neutral, and negative charge states. *Physical Review B* **86**, 085306 (2012).

[21] Lindblad, G. On the generators of quantum dynamical semigroups. *Communications in Mathematical Physics* **48**, 119–130 (1976).

[22] Rudolph, T. Why i am optimistic about the silicon-photonic route to quantum computing. *APL Photonics* **2**, 030901 (2017).

[23] Browne, D. E. & Rudolph, T. Resource-efficient linear optical quantum computation. *Physical Review Letters* **95**, 010501 (2005).

[24] Kambs, B. & Becher, C. Limitations on the indistinguishability of photons from remote solid state sources. *New Journal of Physics* **20**, 115003 (2018).

[25] Pilnyak, Y. *et al.* Simple source for large linear cluster photonic states. *Physical Review A* **95**, 022304 (2017).
